# Ribulose 1,5-Bisphosphate Carboxylase/Oxygenase Is Required in *Bradyrhizobium diazoefficiens* for Efficient Soybean Root Colonization and Competition for Nodulation

**DOI:** 10.3390/plants13172362

**Published:** 2024-08-24

**Authors:** Rocío S. Balda, Carolina Cogo, Ornella Falduti, Florencia M. Bongiorno, Damián Brignoli, Tamara J. Sandobal, María Julia Althabegoiti, Aníbal R. Lodeiro

**Affiliations:** 1 Instituto de Biotecnología y Biología Molecular (IBBM), Facultad de Ciencias Exactas, Universidad Nacional de La Plata (UNLP), Centro Científico Tecnológico (CCT)-La Plata, Consejo Nacional de Investigaciones Científicas y Técnicas (CONICET), La Plata 1900, Argentina; rociobalda@gmail.com (R.S.B.); carolina.cogo@yahoo.com.ar (C.C.); ornefalduti@gmail.com (O.F.); damianbrignoli@biol.unlp.edu.ar (D.B.); tamarajsandobal@gmail.com (T.J.S.); mja@biol.unlp.edu.ar (M.J.A.); 2 Departamento de Ciencias Básicas, Facultad de Ingeniería, UNLP, La Plata 1900, Argentina; 3 Cátedra de Genética, Facultad de Ciencias Agrarias y Forestales, UNLP, La Plata 1900, Argentina; florenciabongiorno@gmail.com

**Keywords:** *Bradyrhizobium*, RuBisCO, CBB pathway, rhizosphere

## Abstract

The Hyphomicrobiales (Rhizobiales) order contains soil bacteria with an irregular distribution of the Calvin–Benson–Bassham cycle (CBB). Key enzymes in the CBB cycle are ribulose 1,5-bisphosphate carboxylase/oxygenase (RuBisCO), whose large and small subunits are encoded in *cbbL* and *cbbS*, and phosphoribulokinase (PRK), encoded by *cbbP*. These genes are often found in *cbb* operons, regulated by the LysR-type regulator CbbR. In *Bradyrhizobium*, pertaining to this order and bearing photosynthetic and non-photosynthetic species, the number of *cbbL* and *cbbS* copies varies, for example: zero in *B. manausense*, one in *B. diazoefficiens*, two in *B. japonicum*, and three in *Bradyrhizobium* sp. BTAi. Few studies addressed the role of CBB in *Bradyrhizobium* spp. symbiosis with leguminous plants. To investigate the horizontal transfer of the *cbb* operon among Hyphomicrobiales, we compared phylogenetic trees for concatenated *cbbL*-*cbbP*-*cbbR* and housekeeping genes (*atpD*-*gyrB*-*recA*-*rpoB*-*rpoD*). The distribution was consistent, indicating no horizontal transfer of the *cbb* operon in Hyphomicrobiales. We constructed a Δ*cbbLS* mutant in *B. diazoefficiens*, which lost most of the coding sequence of *cbbL* and has a frameshift creating a stop codon at the N-terminus of *cbbS*. This mutant nodulated normally but had reduced competitiveness for nodulation and long-term adhesion to soybean (*Glycine max* (L.) Merr.) roots, indicating a CBB requirement for colonizing soybean rhizosphere.

## 1. Introduction

Certain soil bacteria possess the ability to fix CO_2_. Among them are several members of the Hyphomicrobiales order, which encompasses photosynthetic and non-photosynthetic bacterial species. *Bradyrhizobium diazoefficiens* [1] is a non-photosynthetic member of this order, which may thrive under different lifestyles in the soil, including free-living in planktonic or sessile states [2] or as symbionts, fixing atmospheric N_2_ inside nodules of different tropical legumes such as soybean (*Glycine max* (L.) Merr.), cowpea (*Vigna unguiculata* (L.) Walp.), mung bean (*V. radiata* (L.) Wilczek), and siratro (*Macroptilium atropurpureum* (Moc. & Sessé ex DC.) Urb.) [3,4]. To cope with such a diversity of lifestyles, *B. diazoefficiens* has a large genome (>9 Mbp) that encodes a wide menu of metabolic pathways [5]. Among them is the Calvin–Benson–Bassham cycle (CBB), for which the *cbb* operon includes single copies of *cbbL* and *cbbS* encoding the large and small subunits of Type IC ribulose 1,5-bisphosphate carboxylase/oxygenase (RuBisCO) [6], *cbbP* for phosphoribulokinase (PRK) [7], *cbbX* for the RuBisCO activase [8], as well as other genes encoding enzymes of the pentose phosphate pathway (PPP) such as fructose 1,6-bisphosphatase (*cbbF*) [9], transketolase (*cbbT*) [10], fructose-bisphosphate aldolase (*cbbA*) [11], and ribulose-phosphate 3-epimerase (*rpe*) [12]—named here as *cbbE* as it is part of the *cbb* operon [13]. In addition, the LysR-type transcriptional regulator *cbbR* [14] lies at the opposite strand near the 5’ end of the *cbb* operon. Meanwhile, other species of the genus, such as *B. japonicum*, as well as *Bradyrhizobium* sp. CCGE-LA001, ORS 278, and *B. oligotrophicum* S58, possess an additional Type IC RuBisCO, while the photosynthetic strain *Bradyrhizobium* sp. BTAi also possesses a Type IAc RubisCO and an additional *cbb* operon [6]. Surprisingly, there are two *Bradyrhizobium* spp. strains (*B. manausense* BR3351 and *Bradyrhizobium* sp. URHD0069) for which homologs to RuBisCO are absent in their genomic sequences. As in *Bradyrhizobium*, the distribution of these genes in Hyphomicrobiales is not uniform. For instance, members of *Rhizobium*, *Sinorhizobium*, and *Ensifer* possess them, while members of the closely related *Agrobacterium* do not, and in a nearby branch, members of *Mesorhizobium* also possess *cbb* genes while the closely related *Aquamicrobium*, *Aminobacter*, and *Nitratireductor* do not. Since there is evidence that, during evolution, RuBisCO genes were horizontally exchanged among different phyla including prokaryotes, archaea, and higher plants [15,16,17], this diversity in both *cbb* operon structures and photosynthetic capabilities suggests the possibility that, within Hyphomicrobiales, the *cbb* operon might also have been exchanged by horizontal gene transfer between photosynthetic and non-photosynthetic members of this order.

RuBisCO activity was detected in *B. diazoefficiens* cells growing chemoautotrophically in microaerobiosis using H_2_ as electron donor and CO_2_ as carbon source [18]. Under these conditions, *cbbP*, *cbbL*, and *cbbS* are overexpressed in relation to growth in an ambient air atmosphere [19]. In addition, *B. diazoefficiens* chemoautotrophic growth was observed in ambient air using thiosulfate as an electron donor in the absence of an externally added carbon source, and a *cbbL*-defective mutant is unable to grow in these conditions [20]. For heterotrophic growth, *B. diazoefficiens* may use a wide range of carbon sources [1]. Two important carbon sources are D-mannitol and L-arabinose, which follow two different pathways for their preparatory catabolic phases in aerobic conditions [21]. Since *B. diazoefficiens* does not catabolize hexoses or pentoses through the Emden–Meyerhof–Parnas pathway ([21] and references therein), in cultures grown with D-mannitol, the PPP is preferred; in addition, PRK and RuBisCO are synthesized, which suggests that CBB cycle is also at work, whereby this bacterium might add CO_2_ to its total carbon assimilation [21]. Differently, in aerobic cultures grown with L-arabinose, PRK and RuBisCO are not detected, and the first catabolic steps occur by the L-2-keto-3-deoxyarabonate (LKDA) pathway [21] because, with L-arabinose being the enantiomer of D-arabinose, the former cannot enter the PPP at the level of D-ribulose. These results suggest that, for *B. diazoefficiens*, the CBB cycle might play some role in (micro)aerobic environments even in the presence of certain carbon sources and, in particular, it may be important for some process in the soil atmosphere. The symbiotic association of *B. diazoefficiens* with soybean roots involves a preinfection processes characterized by rhizosphere colonization, bacterial adhesion to root surfaces, induction of root hairs deformation, and early root hair penetration. Although there are studies that demonstrated the role of RuBisCO in the symbiosis between *Aeschynomene* plants and photosynthetic bradyrhizobia [22], no studies addressed the possible role of the CBB cycle during the early symbiotic association of *B. diazoefficiens* with soybean roots.

To gain insight about the above-mentioned queries, here we compared the genomic regions containing the *cbb* operon in diverse Hyphomicrobiales and obtained a *B. diazoefficiens* mutant with a deletion in the catalytic region of *cbbL* and a stop codon in the N-terminus of *cbbS* to study its effects on early interaction with soybean plants. No conclusive evidence about horizontal gene transfer of the *cbb* operon in Hyphomicrobiales was obtained. In turn, the mutant strain was impaired in root colonization as well as competition for nodulation, indicating that RuBisCO may be necessary for establishment of rhizobia in the rhizospheric environment.

## 2. Results

### 2.1. Phylogenetic Relationships of Housekeeping Genes and the Cbb Operon in Hyphomicrobiales

**Figure 1:**
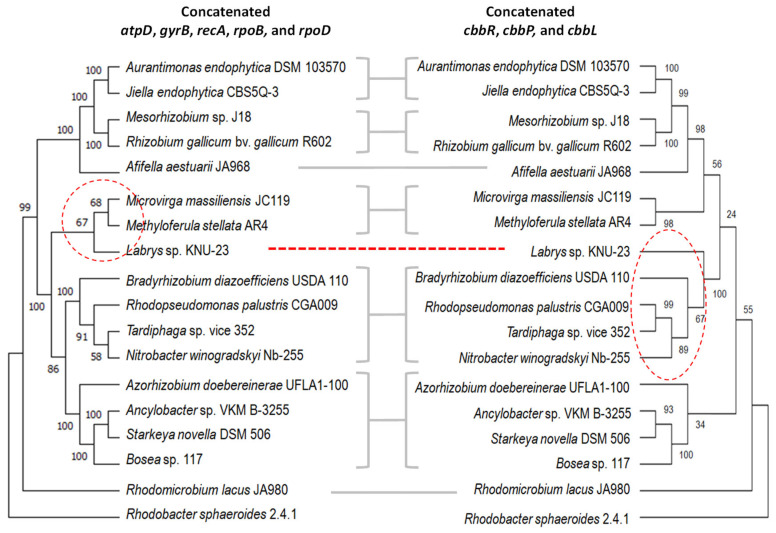
Comparison of the phylogenetic trees obtained with concatenated *atpD*, *gyrB*, *recA*, *rpoB*, and *rpoD* housekeeping genes (**left**) or concatenated *cbbR*, *cbbP*, and *cbbL* as representative of the *cbb* operon (**right**). The position of *Labrys* sp. KNU-23, which differs between both trees, is indicated by dotted red lines. *Rhodobacter sphaeroides* 2.4.1 was used as outgroup. Numbers indicate the percentage of replicate trees in which the associated taxa clustered together in the bootstrap test (1000 replicates).

To compare the phylogenetic relationships of the CBB pathway with those of housekeeping genes, we concatenated *cbbR*, *cbbL*, and *cbbP* on the one hand, and *atpD*, *gyrB*, *recA*, *rpoB*, and *rpoD* on the other, and we compared their distributions among various Hyphomicrobiales. We observed a high degree of consistency between the trees obtained for each group of concatenates, except for *Labrys* sp. KNU-23, which appeared closely related to *Microvirga massiliensis* JC 119 and *Methyloferula stellata* AR4 in the housekeeping tree, while in the *cbb* tree it appeared in the same branch as *B. diazoefficiens* USDA 110, *Rhodopseudomonas palustris* CGA009, *Tardiphaga* sp. vice 352, and *Nitrobacter winogradskyi* Nb-255 (Figure 1). Since this change in the phylogenetic position of the *Labrys* sp. KNU-23 *cbb* operon suggested a horizontal gene transfer event, we looked for possible insertion sequences or tRNA sequences at the ends of this operon and evaluated possible changes in its GC contents with respect to the DNA flanking region. Although we observed a tRNA-Arg at 5 kb upstream *ccbR*, no other putative insertion sequences were found. Moreover, the GC content in this region was not different to that of the flanking regions or the general GC content of this species. Perhaps genomic signatures, if they existed, have been lost over time.

Some of the species analysed in Figure 1 possess a second copy of *cbbL*, although not in the context of an additional *cbb* operon. Therefore, we did not concatenate these extra *cbbL* copies with *cbbP* and *cbbR* because an artifact might be produced by mixing sequences that are in the *cbb* operon with the monocistronic *cbbL* copies. Instead, we performed a second phylogenetic analysis using only *cbbL* and observed that all the *cbbL* monocistronic paralogs grouped together in a separate clade (Supplementary Figure S1). This result indicates that there is considerable sequence divergence between *cbbL* paralogs in each of the species, suggesting that these extra copies could be originated in gene duplications rather than horizontal gene transfer, a hypothesis already considered for evolution of RuBisCO in a wider taxonomic context [15]. In agreement with this hypothesis, in the known example of *Bradyrhizobium* sp. ORS278, the *cbbL* paralog seems inactive [22].

### 2.2. Different Effects of ΔcbbLS Mutation on Early and Late Root Adhesion and Colonization

**Figure 2:**
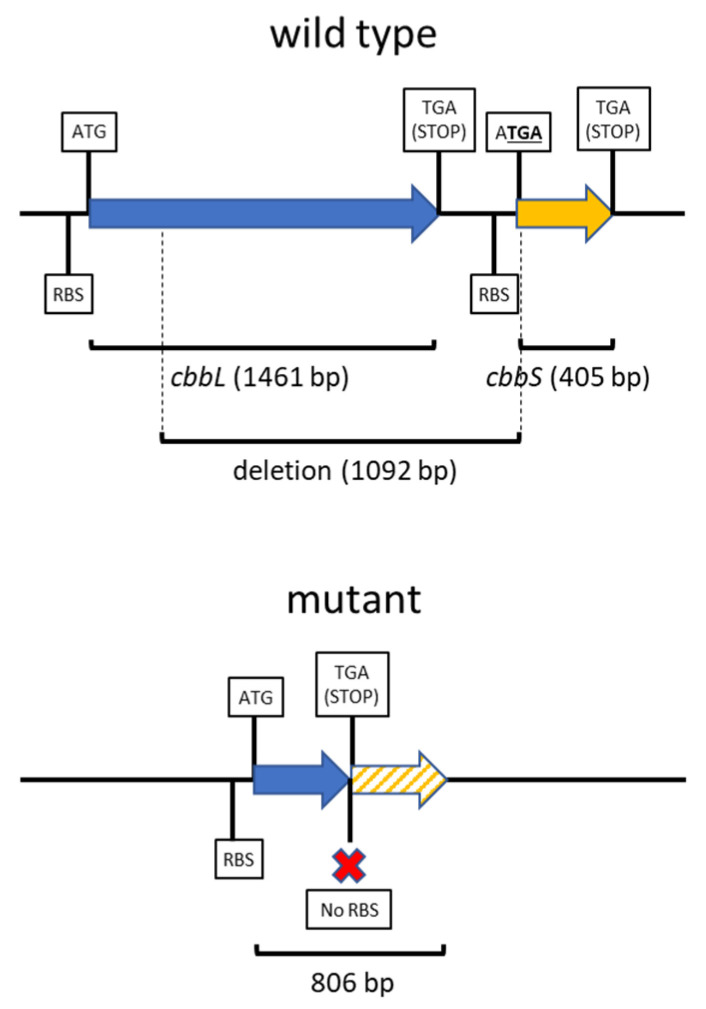
Scheme of the USDA 110 wild-type and Δ*cbbLS* mutant DNA regions. The TGA stop codon that is generated at the 5’ of the *cbbS* ORF is underlined. The wild-type intergenic region between *cbbL* and *cbbS* contains 11 bp (not to scale in the scheme). The 1092 bp deletion eliminated most of the coding region from *cbbL* including its catalytic domain, the *cbbL* stop codon, as well as the *cbbS* RBS. The TGA stop codon generated at the N-terminus of *cbbS* is in-frame with the ATG start codon of *cbbL*, but not in-frame with the *cbbS* ATG start codon. In addition, five additional stop codons were generated downstream (not indicated in the scheme; for details, see supplementary Figure S6). The mutant Δ*cbbLS* gene fragment contains an additional 21 bp segment added by the mutagenesis strategy.

We obtained a deletion mutant in *B. diazoefficiens* USDA 110 by removing 1092 bp encompassing the *cbbL* coding sequence (including the catalytic site) and the 5′-UTR (containing the RBS) of the adjacent *cbbS* gene and introduced a stop codon in the N-terminus of *cbbS* (Figure 2 and Supplementary Materials). Since the coding sequences of the two RuBisCO subunits were affected, we named this mutant Δ*cbbLS*.

**Figure 3:**
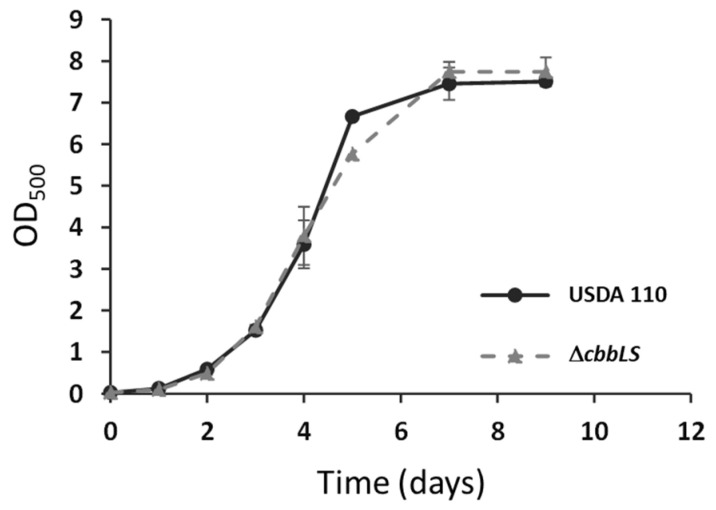
Growth of USDA 110 and Δ*cbbLS* in HMY-Mtl medium. Data are averages ± SD from three independent cultures. When error bars are not shown, they are smaller than the symbol.

Then, we studied the early interaction of this mutant with soybean plants. For these experiments, we obtained *B. diazoefficiens* cells from liquid cultures in HMY-Mtl grown at the exponential phase, which are optimal conditions for *cbb* operon expression, the percentage of viable cells, adhesiveness to plant roots, infectiveness, and competitiveness for nodulation [21,23,24]. In addition, we observed that Δ*cbbLS* and the wild-type USDA 110 grew equally well in liquid HMY-Mtl (Figure 3) whereby differences in adsorption or root colonization described below cannot be attributed to differences in previous inoculum growth.

**Figure 4:**
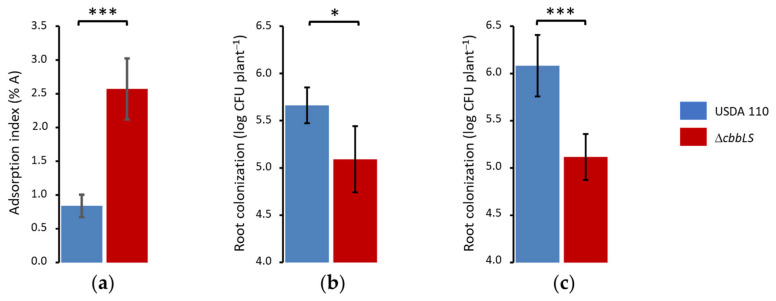
Early interactions of *B. diazoefficiens* USDA 110 and Δ*cbbLS* with soybean (*Glycine max* (L.) Merr.) roots. (**a**) Initial reversible adsorption measured after 1 h incubation; (**b**,**c**) late firm adsorption and rhizosphere colonization, measured after 48 h incubation. In (**b**), bacterial cells were recovered from roots and rhizosphere by shaking at 180 rpm during 5 min. In (**c**), the remaining bacterial cells after the first wash were recovered by vortexing at maximum speed during 2 min. In all graphs, bars represent average from 10 plants (**a**) or 5 plants (**b**,**c**), error bars represent confidence intervals with *p* < 0.05, and asterisks indicate significant differences with *p* < 0.05 (one asterisk) or 0.005 (three asterisks) obtained by one-way ANOVA. All results are representative from at least two independent experiments.

In short-term experiments, we incubated soybean roots in liquid MFS for 1 h with USDA 110 and the Δ*cbbLS* mutant and observed that in these conditions early adsorption of the Δ*cbbLS* mutant was higher than that of the wild type (Figure 4a). This higher adsorption correlated with a higher production of extracellular polysaccharides by the Δ*cbbLS* mutant, which had 6.6 ± 0.6 μg per optical density unit at 500 nm (OD_500_), while the wild type had 4.4 ± 0.2 μg extracellular polysaccharide per OD_500_ unit.

In another series of experiments, we incubated Δ*cbbLS* with the plants for 48 h in vermiculite pots at 28 °C to measure late adsorption and rhizosphere colonization. We measured the colonization of the root environment by detaching bacteria from rhizosphere and roots with a mild wash and firmly adsorbed bacteria by vortexing at maximum speed. In comparison to the wild type, a substantial reduction in the numbers of CFU released was noticed, especially in the fraction corresponding to firm adsorption to root surfaces (Figure 4b,c). Therefore, it seems plausible that, despite a lower initial adsorption, the wild type either achieved higher firm adsorption [25,26] or grew better in the rhizospheric environment than the Δ*cbbLS* mutant leading to better colonization, although these two possibilities are not mutually exclusive.

### 2.3. ΔcbbLS Mutation Affects Competition for Nodulation

**Figure 5:**
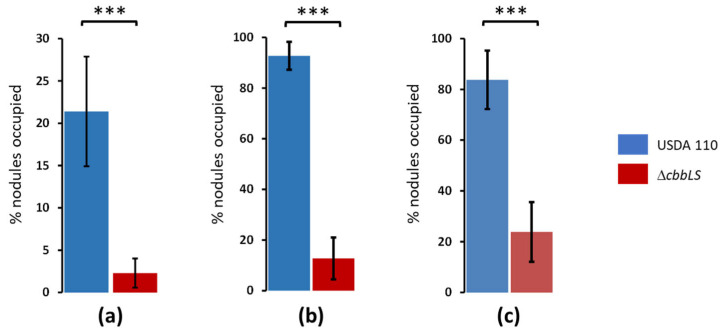
Competition for soybean (*Glycine max* (L.) Merr.) nodulation between *B. diazoefficiens* USDA 110 and Δ*cbbLS* mutants. (**a**) Percentage of nodules occupied by USDA 110 or Δ*cbbLS* in competition against the Sm-resistant LP 3004, a near-isogenic spontaneous derivative from USDA 110; (**b**,**c**) direct competition between USDA 110 and the Δ*cbbLS* mutant clone 1 (**b**) or clone 2 (**c**). Each bar represents the percentage of nodules occupied by the referred strain either as single occupant (**a**) or in single occupation + double occupation together with the other strain (**b**,**c**). In (**a**), nodules occupied by either USDA 110 or the Δ*cbbLS* mutant were recorded as Sm-sensitive, whereby nodules occupied by LP 3004 alone could not be distinguished from those occupied by both competitor strains because all these nodules were Sm-resistant. Therefore, the sum of percentages in this competition is <100%, because it represents the Sm-sensitive nodules only. In (**b**,**c**), USDA 110 and the Δ*cbbLS* mutant were distinguished by PCR, whereby double occupation was recorded, and therefore the sum of percentages is >100% because nodules containing both strains were counted twice, i.e., as occupied by USDA 110 and by the Δ*cbbLS* mutant. The percentage of double occupation was 8.20 ± 6.44% (**b**) or 7.60 ± 5.28 (**c**) (average ± confidence interval with *p* < 0.05). In all graphs, bars represent the average from 10 (**a**) or 8 (**b**,**c**) plants, error bars represent confidence intervals with *p* < 0.05, and asterisks indicate significant differences with *p* < 0.005 obtained by one-way ANOVA. All results are representative from two independent experiments.

To observe if this mutation produced an effect on nodulation, the Δ*cbbLS* strain and the wild-type USDA 110, grown in the same conditions as above, were inoculated each in five soybean plants, which were cultured under hydroponic conditions in pots irrigated with N-free MFS plant nutrient solution for 30 days after inoculation (dai). At this time, plants were removed, roots were washed, and they were observed for nodulation. Both the Δ*cbbLS* mutant and the wild type produced around 20 morphologically normal nodules per plant, without differences in nodules’ positions or sizes (Supplementary Figure S2). There were also no differences in shoot dry weight (around 470 mg plant^−1^) or chlorophyll contents (around 31 SPAD units plant^−1^) between USDA 110 and the Δ*cbbLS* mutant. However, when each of these strains was coinoculated in a 1:1 proportion with LP 3004—the nearly isogenic Sm-derivative from USDA 110 [27,28]—the wild type showed 10-fold higher nodule occupancy than that of the mutant strain at 30 dai, despite LP 3004 being consistently more competitive (Figure 5a).

In these experimental conditions, we were unable to distinguish nodules occupied by only LP 3004 from those occupied by both competitor strains together since both kinds of bacterial samples contained LP 3004 and therefore both grew in the presence of Sm. To assess double occupation, in separate experiments we measured direct competition between *B. diazoefficiens* USDA 110 and the Δ*cbbLS* mutant, by coinoculating both strains in a 1:1 proportion and carrying out the experiment as described above, and after obtaining the bacteria from the nodules we determined nodule occupation by PCR using the FwUp_RBC and RvDw_RBC primers (Table 1). In this way, the nodules occupied by USDA 110 rendered a single 1692 bp band, those occupied by the Δ*cbbLS* mutant rendered a single 621 bp band, and those occupied simultaneously by both strains (double occupation) rendered both bands. We observed that double nodule occupancy was around 8%, which is similar to values observed previously [27] and corroborated that USDA 110 occupied almost 10 times more nodules than each of the Δ*cbbLS* mutant clones (Figure 5b,c). In all these experiments, control plants inoculated with each strain alone formed nodules occupied by only the inoculated strain, while no nodules were observed in the uninoculated controls.

Together, the similar nodulation achieved with each strain inoculated alone, and the 10-fold reduction in nodules occupation by the mutant strain under competition indicated that, although the Δ*cbbLS* mutant is able to carry out the complete infection and nodulation process, it is less efficient at some early step of the interaction. Our results above indicated that this step may be related to colonization of soybean roots.

## 3. Discussion

The *cbb* operon is irregularly distributed among Hyphomicrobiales, making it difficult to appreciate the importance of the CBB cycle for adaptation of these bacteria to soil conditions. The genus *Bradyrhizobium*, within Hyphomicrobiales, is no exception, since some members such as *B. diazoefficiens* possess only one copy of the *cbb* operon, others such as the photosynthetic *Bradyrhizobium* sp. BTAi possess two, and others still such as *B. manausense* do not have any. This diversity suggests the existence of horizontal gene transfer events among these bacteria, but our comparison of the phylogenetic relationships between three CBB key genes of *cbb* operon with that of housekeeping genes indicated a strong correlation between both phylogenies. The exception to this rule was *Labrys* sp. KNU-23. This genus was first isolated from silt of Lake Mustijärv in Estonia by Vasil’eva and Semenov in 1984 [29], and since then, different *Labrys* species have been isolated and described from different parts of the world; notably, several strains were isolated from legume nodules [30,31] or plant tissues [32], sharing the environment with members of *Bradyrhizobium* and relatives. Therefore, these close contacts might favour the exchange of genetic material among bacterial cells. In addition, we found a sequence signature upstream of *Labrys* sp. KNU-23 *cbb* operon that might indicate horizontal gene transfer, although evidence is tenuous.

The *cbb* operon in *B. diazoefficiens* USDA 110 seems regulated by *cbbR*, as in the close relative *Rhodopseudomonas palustris* [14,33], although the inducer of CbbR is still unknown (Supplementary Figure S3). We observed that the *cbb* operon is induced in liquid aerobic cultures with D-mannitol as a carbon source, but not with L-arabinose as a carbon source, indicating some metabolic specificity in the conditions under which the CBB cycle is required in an ambient air atmosphere [21]. Since the early preinfection events in the *B. diazoefficiens*–soybean symbiosis elapses in the soil atmosphere, we wondered if the CBB cycle might play some role at this stage of the symbiotic interaction by characterizing root colonization and nodulation of the *B. diazoefficiens* Δ*cbbLS* mutant. In short-time incubations, the Δ*cbbLS* mutant had a higher level of early adsorption than that of the wild type, but in longer term incubation, the Δ*cbbLS* mutant was impaired in root colonization. Adsorption of rhizobia to plant roots was characterized as a two-step process: an early and reversible adsorption occurs in the first few hours of contact between roots and bacteria, followed by a late and irreversible adsorption past 12 h or more [25]. These different adsorption steps seem mediated by different adhesin molecules [26,34]. Therefore, our results indicate that the Δ*cbbLS* mutant was differently affected for these kinds of adsorption. In this regard, the behaviour at early adsorption correlated with extracellular polysaccharide levels, which is known to affect this early preinfection step [24]. As a non-exclusive alternative, we may consider that growth in the rhizosphere may have been hampered in the Δ*cbbLS* mutant.

In another series of experiments, the Δ*cbbLS* mutant strain nodulated soybean plants normally and produced the same numbers of nodules as the wild type at vegetative stage. The nodules produced by the mutant displayed an external morphology alike functional nodules, with similar distribution along the roots as wild-type nodules, indicating that the infection and nodulation processes were not altered by the mutation. In agreement with these observations, *cbbL* or *cbbS* transcripts or gene products were not detected in wild-type *B. diazoefficiens* bacteroids obtained from active soybean nodules [35,36,37] and *cbbS* transcripts were found only in bacteroids from senescent soybean nodules [37], indicating that RuBisCO is not required for the symbiotic lifestyle in *B. diazoefficiens*.

Differently from nodulation by each strain inoculated alone, when the Δ*cbbLS* mutant was coinoculated with the wild-type strain, the former occupied very few nodules although all nodules were normal again, indicating that some preinfection process was affected by the mutation, in agreement with our observation that Δ*cbbLS* was affected in its ability to colonize the soybean roots. Taken together, all these results indicate that, in *B. diazoefficiens*, RuBisCO should be necessary only in the free-living state before infection, as well as after abandoning the senescent nodule to reinitiate a new cycle of soil and rhizosphere colonization.

In general, bacteria thriving in the rhizosphere benefit from the nutrient richness found there, which comes from passive and active processes of exudation from the plant roots. The composition of root exudates is rather complex and includes aminoacids, organic acids, carbohydrates, secondary metabolites, and larger molecules such as polysaccharides, proteins, and glycoproteins, some of which are involved in molecular signal exchanges between rhizobia and the plant symbiont [38,39,40,41,42,43,44]. In these studies, the presence of arabinose was detected in soybean root exudates, although it is not specified whether it is L-arabinose or D-arabinose, which is an important distinction because, while L-arabinose is catabolized through the LKDA pathway and induces the synthesis of lateral flagella in *B. diazoefficiens* [21], D-arabinose does not induce the synthesis of these flagella [45], and, according to KEGG metabolic charts for *B. diazoefficiens*, this isomer is probably catabolized by the PPP-CBB cycle. In addition, the presence of D-mannitol in soybean root exudates was never reported—which is intriguing because this polyol is one of the best C-sources for *Bradyrhizobium* spp.—although other sugars that may be catabolized similarly by this bacterium, such as D-glucose or D-fructose [1], are commonly observed in root exudates. Nevertheless, chemical detection and identification of all the molecular components of root exudates is difficult, and in addition, root exudate composition varies along root position, time, and according to plant status [46,47,48,49].

In contrast to *B. diazoefficiens*, it was shown that the photosynthetic *Bradyrhizobium* sp. ORS278 requires RuBisCO for efficient N_2_ fixation in symbiosis with *Aeschynomene indica* L. [22]. This bacterium possesses two copies of *cbbL* and *cbbS*, one set in the canonical *cbb* operon and the others at a different location in the genome, which were named 1 and 2, respectively. However, only *cbbLS1* is expressed in *A. indica* nodules. A Δ*cbbL1* mutant is severely affected by N_2_ fixation in symbiosis with these plants but is not affected at all in N_2_ fixation in vitro, which indicates that the effect of RuBisCO inactivation is not directly related to nitrogenase activity. Based on previous studies on photosynthetic bacteria, the authors proposed that during a precise time interval between decrease in oxygen concentration in the nodule and the onset of nitrogenase activity, an excess of reducing power might build up from electrons produced at the final steps of the oxidation of plant-supplied organic carbon, thus leading to imbalance of redox potential. To overcome this situation, the CBB cycle might serve as sink of reducing power excess, and therefore the absence of RuBisCO activity might indirectly preclude N_2_ fixation by producing a metabolic disturbance in the nodule [22]. In the case of the non-photosynthetic *B. diazoefficiens*, such disturbance might take place outside the nodule. However, it should be noted that both the wild type and the Δ*cbbLS* mutant grew equally well in HME-Mtl. Because, in our experiments, soybean plantlets were inoculated with HME-Mtl-grown rhizobia, it seems that, if an excess of reducing power is built up in the rhizosphere, root exudates should provide some electron donors in addition to the carbon sources. However, the possible presence of such additional electron donors was not reported at that time in the soybean root exudates.

In the rhizosphere environment, CO_2_ concentration is high, especially around actively growing roots, due to the combined effects of root respiration, hampered diffusion of CO_2_ in water, and air confinement in the soil pores [50,51]. Therefore, CO_2_ fixation might be an advantageous trait for bacteria that colonize and associate to plant roots. Hence, the role of RuBisCO (and PRK) in the rhizosphere might be more related to CO_2_ fixation to harness the high CO_2_ concentration around actively growing roots, where the points of entry by infection threads are located. However, more experimental evidence is required to proof this hypothesis, such as direct measurements of CO_2_ consumption by the wild type and the mutant in the rhizosphere. If this is the case, an attractive possibility is that inoculants based on *B. diazoefficiens* grown with D-mannitol might sequestrate CO_2_ in the rhizosphere, thus making an additional contribution to sustainable agriculture, beyond its largely known role as N_2_-fixer. Field experiments with measurements of CO_2_ evolution in the soil surface are required to assess this possibility.

## 4. Materials and Methods

### 4.1. Bacterial Strains and Culture Conditions

*Bradyrhizobium diazoefficiens* USDA 110 (wild type, with natural resistance to chloramphenicol) [1], LP 3004 (spontaneous streptomycin-resistant nearly isogenic derived from USDA 110) [27,28], and Δ*cbbLS* (mutant derived from USDA 110, see below) were stored in yeast extract mannitol (YM) [52] supplemented with 20% (*v*/*v*) glycerol at −80 °C. For solid culture or colony-forming unit (CFU) counting, we supplemented YM with 1.5% agar (YMA). For plant assays, we grew liquid cultures in HM salts–yeast-extract medium (HMY) supplemented with 5 g L^−1^ D-mannitol (HMY-Mtl) at 30 °C with rotary shaking at 180 rpm [21]. For conjugation, we grew *B. diazoefficiens* in peptone-salts-yeast extract (PSY) [53] and *Escherichia coli* DH5α and S17-1 in Luria–Bertani (LB) [54]. Antibiotics were used at the following concentrations (mg L^−1^): streptomycin (Sm), 400 (*B. diazoefficiens*); kanamycin (Km), 150 (*B. diazoefficiens*) or 25 (*E. coli*); chloramphenicol (Cm), 20 (*B. diazoefficiens*).

### 4.2. Construction of the ΔcbbLS Mutant

We carried out general cloning procedures essentially as described [54]. For mutagenesis, we proceeded essentially as described [55]. To delete a 1092 bp fragment containing the *cbbL* coding sequence and the 5′-UTR of the adjacent *cbbS* from the *B. diazoefficiens* USDA 110 genome, we proceeded as follows: We amplified the fragments Up_RBC (encompassed by primers FwUp_RBC and RvUp_RBC) and Dw_RBC (encompassed by primers FwDw_RBC and RvDw_RBC) by PCR (Table 1). We designed the primers in order to possess 21 nt complementary sequences at the Up_RBC-3′ and Dw_RBC-5′ ends, in such a way that they may form a single fragment to be amplified with the FwUp_RBC and RvDw_RBC primers, giving rise to the 621 bp UpDw_RBC fragment (Supplementary Figure S4). In addition, we designed a *Bam*HI site at the 21 bp joint between Up_RBC and Dw_RBC. We purified this fragment and inserted it in the pK18*mobsacB* suicide vector with *Eco*RI and *Hin*dIII, whose restriction sequences were incorporated in the 5′ ends of FwUp_RBC and RvDw_RBC primers (Table 1). In this way, we obtained the pCC1 plasmid, which was electroporated into *E. coli* DH5α. After selection of transformants carrying pCC1 in Km, we purified the plasmid and checked the correct insertion of the UpDw_RBC fragment by PCR with M13 primers and plasmid digestion (Supplementary Figure S5). Then, we electroporated the pCC1 plasmid into *E. coli* S17-1 and transferred it to *B. diazoefficiens* USDA 110 by biparental mating as described [55]. Next, we selected single crossing-over *B. diazoefficiens* recombinants (Supplementary Figure S6) by plating in the presence of Cm and Km, and in a second step, we selected double crossing-over recombinants (Supplementary Figure S7) by plating in the presence of 10% (*w*/*v*) sucrose and confirming that the sucrose-resistant selected clones were Km-sensitive. Since we observed that the first four nucleotides in the *cbbS* coding sequence are ATGA, we planned the FwUp_RBC and RvUp_RBC primers so as to amplify the first 380 bp of *cbbL* and link it to the first bp of *cbbS* in order to produce a frameshift that replaces the ATG start codon of *cbbS* by a TGA stop codon (Supplementary Figure S8). In doing so, we observed that five additional stop codons were introduced in the frame-shifted *cbbS* sequence. We confirmed these mutations in the *B. diazoefficiens* derivative strain by sequencing the entire 818 bp region using the FwExt_RBC and RvExt_RBC primers (Table 1 and Supplementary Figure S6). Sequencing was carried out at Macrogen Inc. (Seoul, Republic of Korea). This mutant is referred as Δ*cbbLS*.

### 4.3. Sequence Comparisons and Construction of Phylogenetic Trees

We compared the amino acid sequence of CbbL from *B. diazoefficiens* USDA 110 to the BLASTP database [56], excluding the *Bradyrhizobium* genus and including only Hyphomicrobiales members. We set alignment parameters to e-value = 0.0005, BLOSUM62 matrix, and a maximum of 250 targets. From the sequences obtained, we chose those included in *cbb* operons and with higher identity to *B. diazoefficiens* USDA 110, then filtered them in such a way to avoid repeated species as well as those labelled as “multispecies”. When a species was repeated in the list of sequences, we picked those sequences with the best match to *B. diazoefficiens* USDA 110 CbbL. In addition, we chose *Rhodobacter sphaeroides* as outgroup. Then, we included CbbP and CbbR sequences from the strains chosen in the first step in a concatenated CbbR-CbbP-CbbL sequence, which we submitted to a MEGA X analysis [57] to obtain the multiple alignment and to construct the phylogenetic tree using a neighbour-joining statistical analysis [58]. In parallel, we carried out this procedure with these same strains using a concatenated sequence of the housekeeping proteins AtpD, GyrB, RecA, RpoB, and RpoD. In a separate analysis, we compared only CbbL, but while including all paralogs in the same set of species.

### 4.4. Plant Assays

Soybean seeds SYN4x1RR were kindly provided by Syngenta Argentina, Vicente López, Argentina. We surface-sterilized and germinated the seeds in water agar 1.5% (*w*/*v*) as described [23,24]. For inoculation, we cultured *B. diazoefficiens* in liquid HMY-Mtl until exponential phase, evaluated by optical density at 500 nm (OD_500_) and CFU counts.

To evaluate early adsorption, we incubated the bacteria with 10 roots for 1 h as described [23]. After washing the soybean roots, we quantified the adsorbed bacteria by releasing them from the roots by shaking them separately in N-free modified Fåhraeus plant nutrient solution (MFS) [59] and counting the remaining bacteria adsorbed to each root by counting the developed microcolonies as described [23,33]. From these counts, we obtained the adsorption index (*%A*) by relating the total number of bacteria adsorbed, expressed as percentage of the total number of bacteria inoculated [23].

To evaluate rhizosphere colonization, we surface-sterilized seeds and germinated them in water agar as above, and when rootlets reached 2–3 cm in length, we planted them in 5 sterile vermiculite pots per condition at 1 plant per pot, which were irrigated with MFS to field capacity. We inoculated each plant with 1 mL of each *B. diazoefficiens* strain separately, which were grown in the same conditions already described, capped the pots with sterile film, and left the pots at 28 °C in the dark during 48 h. We then removed the plants from each pot, cut the roots, and immersed each one in separate 50 mL Falcon tubes containing 10 mL of sterile MFS. We agitated the tubes in a rotary shaker at 180 rpm for 5 min and took samples from the MFS supernatants, diluted them, and plated them in YMA. Afterward, we carefully removed roots, blotted each one for a few seconds on sterile paper towels, and suspended each root in a new Falcon tube containing 10 mL fresh sterile MFS. We vortexed this second suspension at maximum speed for 2 min and, again, obtained samples from the MFS supernatant of each tube, diluted them, and plated them in YMA. We counted CFU in 10 replicas from each sample to evaluate the mild and tight adsorption to roots, respectively. In all these experiments, we inoculated the same number of plants with MFS without bacteria as negative controls.

For nodulation assays, we cultivated one plant per 500 mL pot containing sterile vermiculite watered with MFS at planting. Each pot received one pregerminated seedling, the number of pots per strain being indicated for each experiment in the Results section. We inoculated at planting with *B. diazoefficiens* USDA 110 or Δ*cbbLS* cultured in the same conditions as above. We grew the plants in the greenhouse at 30 °C/20 °C day/night temperature and provided watering as required, in sequences comprising two irrigations with sterile distilled water followed by one with sterile MFS. At thirty dai, we removed the plants, observed the nodules external morphology, and counted the nodules.

We measured competition for nodulation as described [57] using *B. diazoefficiens* LP 3004 as reference strain, since it is a Sm-resistant almost-isogenic derivative from USDA 110 [27,50]. Each strain, namely USDA 110 or Δ*cbbLS*, were competed against LP 3004 in 1:1 mixed inocula, prepared as described above. Before mixing, we diluted culture samples so as to set their OD_500_ at the same value for all strains, and then we carried out appropriate dilutions in MFS to set the initial CFU of each sample to approximately 10^6^·mL^−1^. We corroborated the actual numbers of CFU·mL^−1^ by colony counting 10 replicas per sample in YMA. As controls, we inoculated sets of pots with each strain separately, or with sterile MFS without bacteria. We performed inoculation by irrigating the pots with the bacterial suspensions as described [60]. Plants were cultivated as described above and collected at 30 dai. At this time, we removed nodules, surface-sterilized them, extracted the bacteria from the interior of each nodule, and cultured the bacterial extracts in YMA replica plates with or without 400 mg·L^−1^ Sm. Finally, we calculated the proportion of nodules containing antibiotic-sensitive bacteria for each plant as a measure of competitiveness. In these experimental conditions, we were unable to distinguish nodules occupied by only LP 3004 from those occupied by both competitor strains since both kinds of bacterial samples grew in the presence of Sm. To distinguish nodules occupied by both strains simultaneously, we coinoculated USDA 110 with each of two different clones of the Δ*cbbLS* mutant and determined nodules occupancy by PCR using the FwUp_RBC and RvDw_RBC primers as described above.

We measured chlorophyll contents in leaves and shoot dry weights as described [61].

All the above-described experiments were carried out twice in their entirety, and results from one representative independent experiment are shown.

### 4.5. Extracellular Polysaccharide Determinations

We measured exopolysaccharide and capsular polysaccharide contents from bacterial cultures with anthrone as described [24], using glucose as standard.

## Figures and Tables

**Table 1:** Primers used in this study. The restriction sites introduced in the primers are indicated in bold, and the 21 nt fragments introduced to generate complementary sequences between RvUp_RBC and FwDw_RBC are underlined.

Primer	Restriction Site	Sequence
FwUp_RBC	*Eco*RI	AAAA**GAATTC**GCTATTGGGAGCCCGACTAC
RvUp_RBC	*Bam*HI	GCCGTCGAC**GGATCC**GAGGCAAACACGTTGCCGATGATCG
FwDw_RBC	*Bam*HI	TGCCTC**GGATCC**GTCGACGGCATGAAACTGACCCAGGGCTG
RvDw_RBC	*Hin*dIII	AAAA**AAGCTT**AAGGAGATTCGCACCGACTC
FwExt_RBC		GCCGCCTAGTCATTCAGAGA
RvExt_RBC		ACGGTGGTGTAGCGGATTG
M13 Fw		GTAAAACGACGGCCAGT
M13 Rv		GCGGATAACAATTTCACACAGG

## Data Availability

The raw data supporting the conclusions of this article will be made available by the authors on request.

## Supplementary Materials

The following supporting information can be downloaded at https://www.mdpi.com/article/10.3390/plants13172362/s1, Figure S1: Phylogenetic analysis of CbbL sequences, Figure S2: nodules and symbiotic parameters from plants inoculated with the wild type or the Δ*cbbLS* separately, Figure S3: Structure and regulation of the *cbb* operon, Figure S4: Schematic diagram of the strategy used to clone the UpDw_RBC fragment into pK18*mobsacB*, Figure S5: Plasmid pCC1 containing the UpDw_RBC fragment, Figure S6: Single crossing-over insertion of pCC1 into the *B. diazoefficiens* USDA 110 *cbbL* genomic region, Figure S7: Double crossing-over insertion of the fragment UpDw_RBC into the *B. diazoefficiens* USDA 110 *cbbL* genomic region, Figure S8: DNA and protein sequences of the Δ*cbbLS* mutation.
