# Supplementary material for: Ribulose 1,5-Bisphosphate Carboxylase/Oxygenase Is Required in Bradyrhizobium diazoefficiens for Efficient Soybean Root Colonization and Competition for Nodulation
Source: Plants (Basel). 2024 Aug 24;13(17):2362. doi: 10.3390/plants13172362 (PMC11397080; doi:10.3390/plants13172362)
Supplement: Supplementary file 1 [file plants-13-02362-s001.zip › plants-3089206-supplementary.pdf]

Ribulose-1, 5-bisphosphate carboxylase-oxygenase is required in *Bradyrhizobium diazoefficiens* for efficient soybean root colonization and competition for nodulation

Supplemental Material

Rocío S. Balda[1♣], Carolina Cogo[1,2♣♦], Ornella Falduti[1], Florencia M. Bongiorno[3], Damián Brignoli[1,3], Tamara J. Sandobal[1,3], M. Julia Althabegoiti[1], Aníbal R. Lodeiro[1,3*]

[1] Instituto de Biotecnología y Biología Molecular (IBBM), Facultad de Ciencias Exactas, UNLP y CCT-La Plata, CONICET. La Plata, Argentina; ibbm@biol.unlp.edu.ar
[2] Departamento de Ciencias Básicas, Facultad de Ingeniería, UNLP, La Plata, Argentina; cienciasbasicas@ing.unlp.edu.ar
[3] Cátedra de Genética, Facultad de Ciencias Agrarias y Forestales, UNLP, La Plata, Argentina; dcbiologicas@agro.unlp.edu.ar
* Correspondence: lodeiro@biol.unlp.edu.ar
♣ Contributed equally to the work (in alphabetic order)
♦ Present address: Comisión Nacional de Energía Atómica (C.N.E.A.), San Carlos de Bariloche, Argentina

Contents:

Figure S1: Phylogenetic analysis of CbbL sequences**.**
Figure S2: Nodules and symbiotic parameters from plants inoculated with the wild type or the Δ*cbbLS* separately
Figure S3: Structure and regulation of the *cbb* operon.
Figure S4: Schematic diagram of the strategy used to clone the UpDw_RBC fragment into pK18mobsacB.
Figure S5: Plasmid pCC1 containing the UpDw_RBC fragment.
Figure S6: Single crossing-over insertion of pCC1 into the *B. diazoefficiens* USDA 110 *cbbL* genomic region.
Figure S7: Double crossing-over insertion of the fragment UpDw_RBC into the *B. diazoefficiens* USDA 110 *cbbL* genomic region.
Figure S8: DNA and protein sequences of the Δ*cbbLS* mutation.

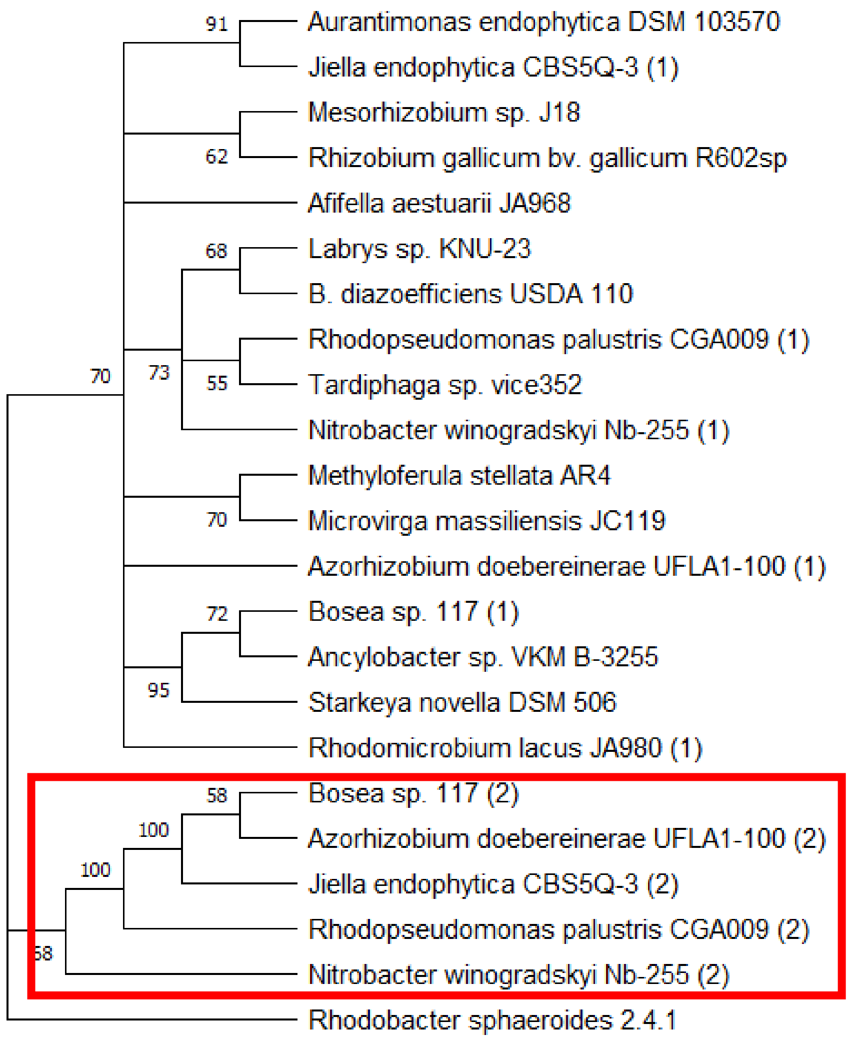

Figure S1 Phylogenetic tree obtained with CbbL copies from the indicated genomes. Paralogs numbered 1 are encoded in canonical *cbb* operons, while those labeled 2 are monocistronic copies laying elsewhere in the genome (squared in red). *Rhodobacter sphaeroides* 2.4.1 was used as outgroup. Numbers indicate the percentage of replicate trees in which the associated taxa clustered together in the bootstrap test (1000 replicates).

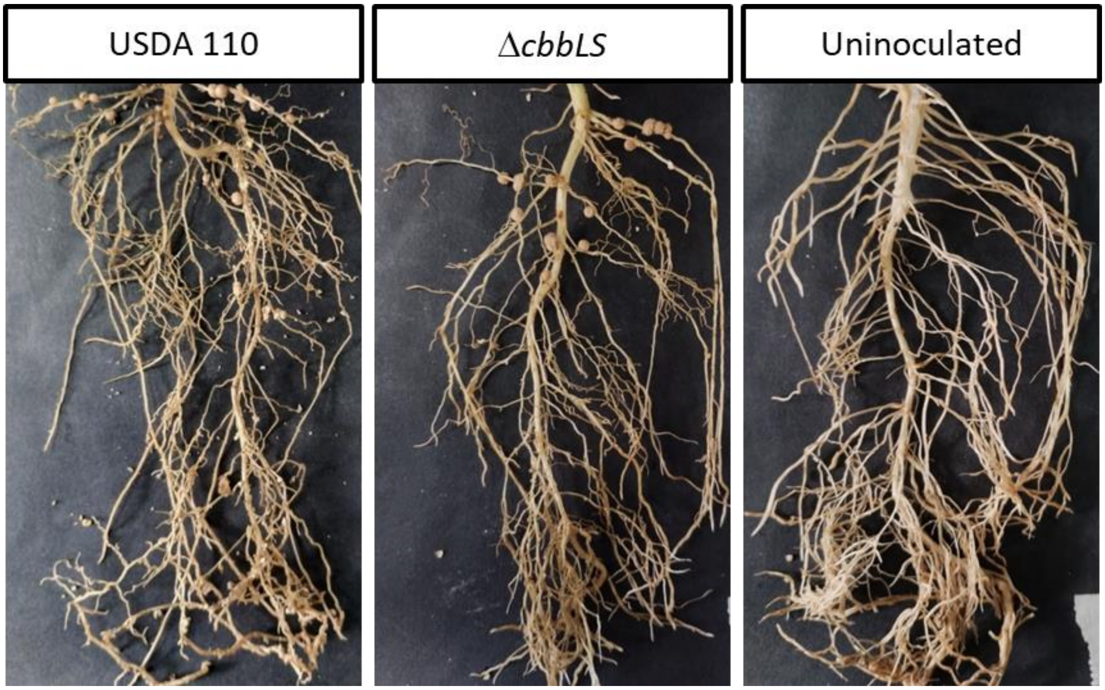

Symbiotic parameters (average ± confidence interval; α=0.05; *n*=5) from plants inoculated with each strain separately

Strain	Number of nodules	Shoot dry weight (mg)	Chlorophyll (SPAD units)
**USDA 110**	23.2 ± 7.0	450 ± 60	32.1 ± 1.9
ΔcbbLS	18.8 ± 1.9	500 ± 110	30.7 ± 0.6
**Uninoculated**	0	470 ± 110	27.3 ± 2.8

Figure S2. Nodules produced by soybean plants inoculated with each strain separately (upper panel) and symbiotic parameters from these plants (lower panel).

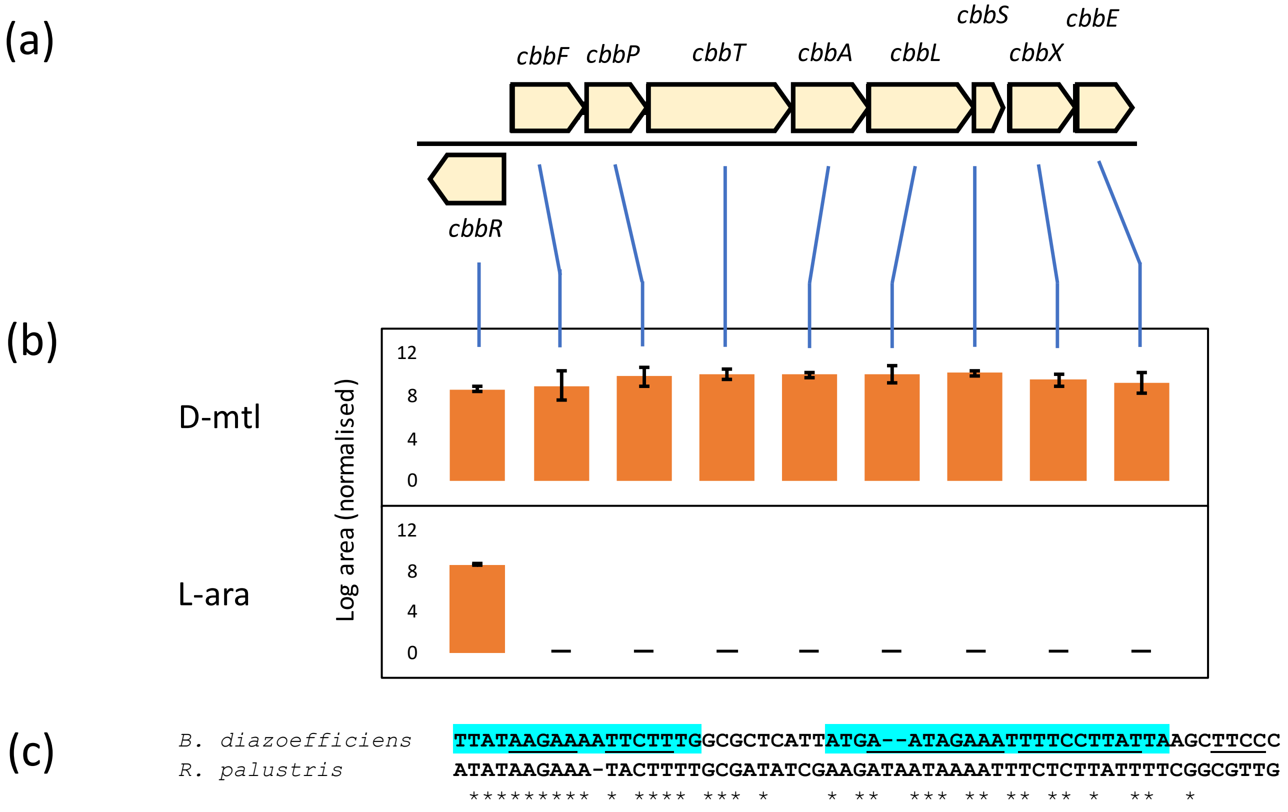

Figure S3. Structure and regulation of the *cbb* operon. **(a)**: schematic representation of the *cbb* operon of *B. diazoefficiens* USDA 110, obtained from MicrobesOnline (http://www.microbesonline.org/). **(b)**: abundance of the encoded polypeptides in HMY medium with D-mannitol (D-mtl) or L-arabinose (L-ara), estimated as average normalised area ± standard deviation of the peptide ion abundances. Hyphens represent absence of the polypeptide in the given condition (calculations performed from data by Cogo *et al.* [21]). **(c)**: Sequence of the 5' region upstream of *cbbF* in *B. diazoefficiens* USDA 110, compared with a similar sequence from *R. palustris*. The binding regions of *cbbR*, as experimentally determined in *R. palustris* [33] are indicated in the *B. diazoefficiens* sequence by light blue shadowing. Underlined are inverted repeats characteristic of LysR-type transcriptional regulators binding motif.

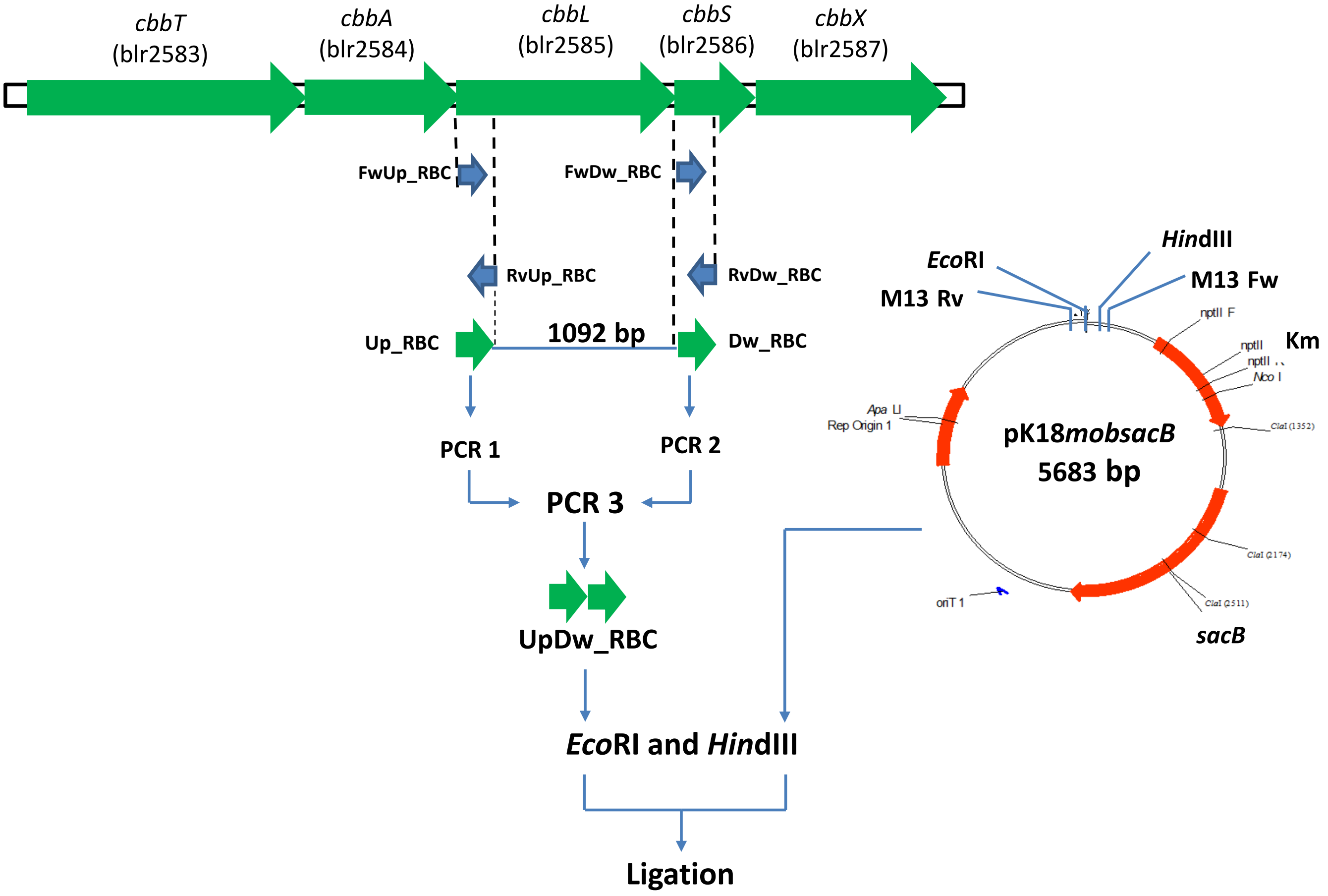

Figure S4. Schematic diagram of the strategy used to clone the UpDw_RBC fragment into pK18*mobsacB*. PCR primers are indicated in blue, while genes and PCR fragments are indicated in green. The map of pK18*mobsacB* shows the relevant sites of the MCS.

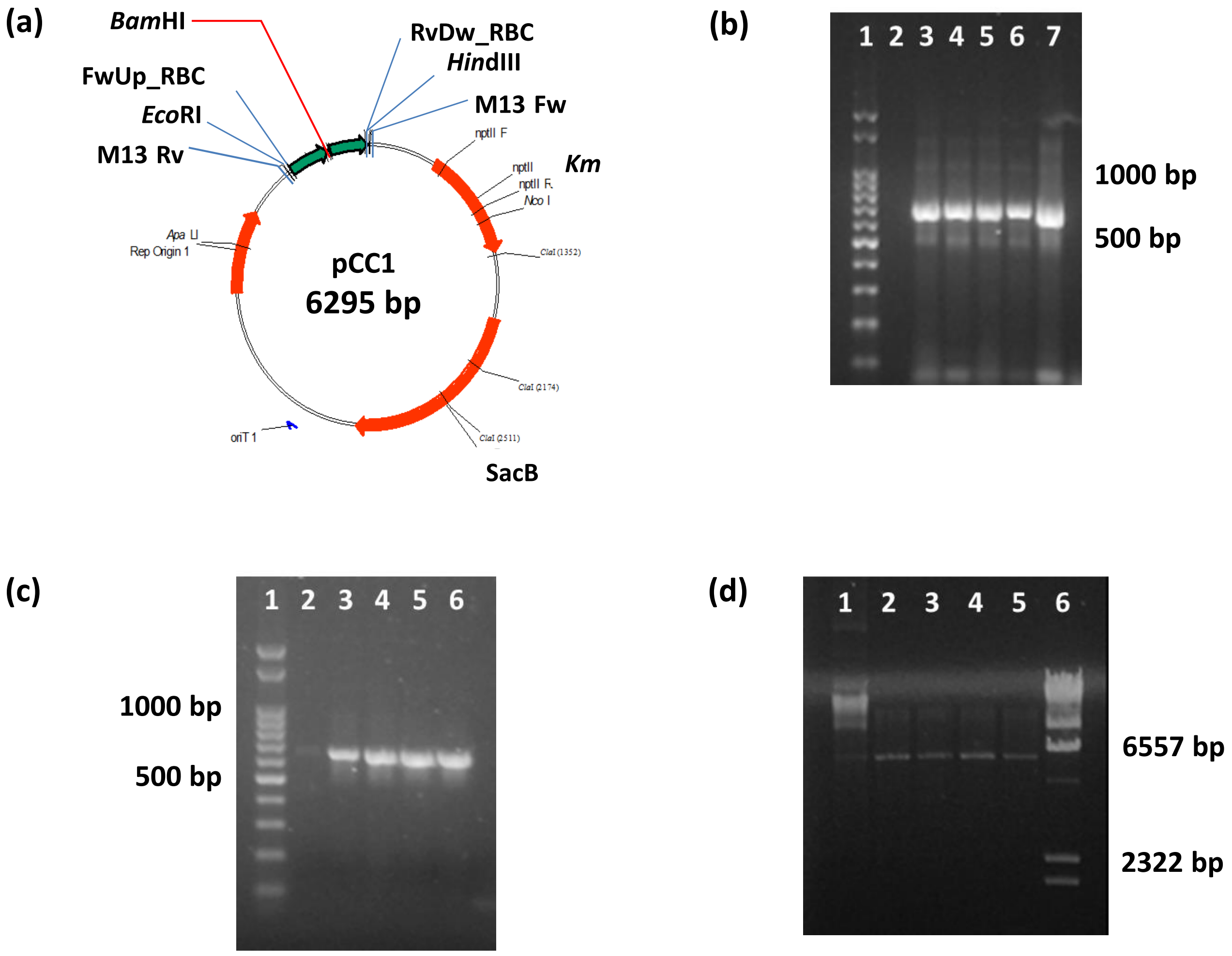

Figure S5. Plasmid pCC1 containing the UpDw_RBC fragment. (a) Scheme of the plasmid. (b) PCR with primers M13 Fw and M13 Rv (c) PCR with primers FwUp_RBC and RvDw_RBC. In both PCR: lane 1, MW markers; lane 2, negative control; lanes 3-7, five Km^r-clones. (d) Digestion of pCC1 with *Bam*HI. Lane 1, non-digested plasmid; lanes 2-5: four Km^r-clones digested with *Bam*HI; lane 6: MW marker. In the three gels, the positions of the relevant MW marker bands are indicated.

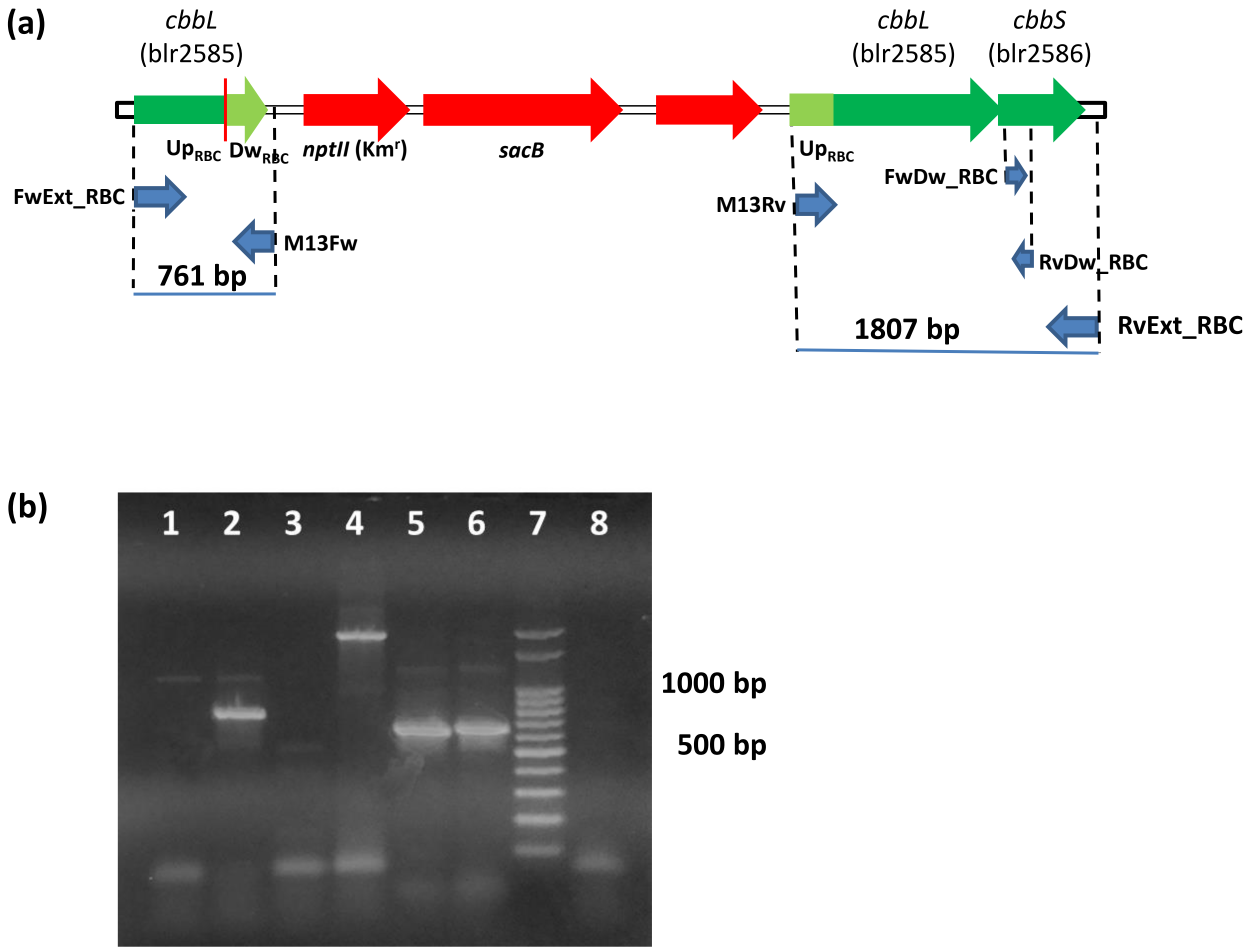

Figure S6. Single crossing-over insertion of pCC1 into the *B. diazoefficiens* USDA 110 *cbbL* genomic region. (a) Scheme of the insertion, showing the recipient *cbb* gene regions in dark green, the plasmid *cbb* gene regions in light green, the plasmid genes in red, the PCR primers in blue, and the *Bam*HI restriction site as a vertical red line. (b) PCR with primers FwExt_RBC and M13 Fw (lanes 1 and 2), M13 Rv and RvExt_RBC (lanes 3 and 4) or FwUp_RBC and RvDw_RBC (lanes 5 and 6). Lane 7, MW markers, with the positions of the relevant MW marker bands indicated at the right. Lane 8: negative control. In lines 2, 4, and 6, a clone with the single insertion in the "down" region of *cbbL* as depicted in panel (a) is shown, while in lines 1, 3, and 5, a clone with the single insertion in the "up" region of *cbbL* is shown (confirmation of this insertion is not shown).

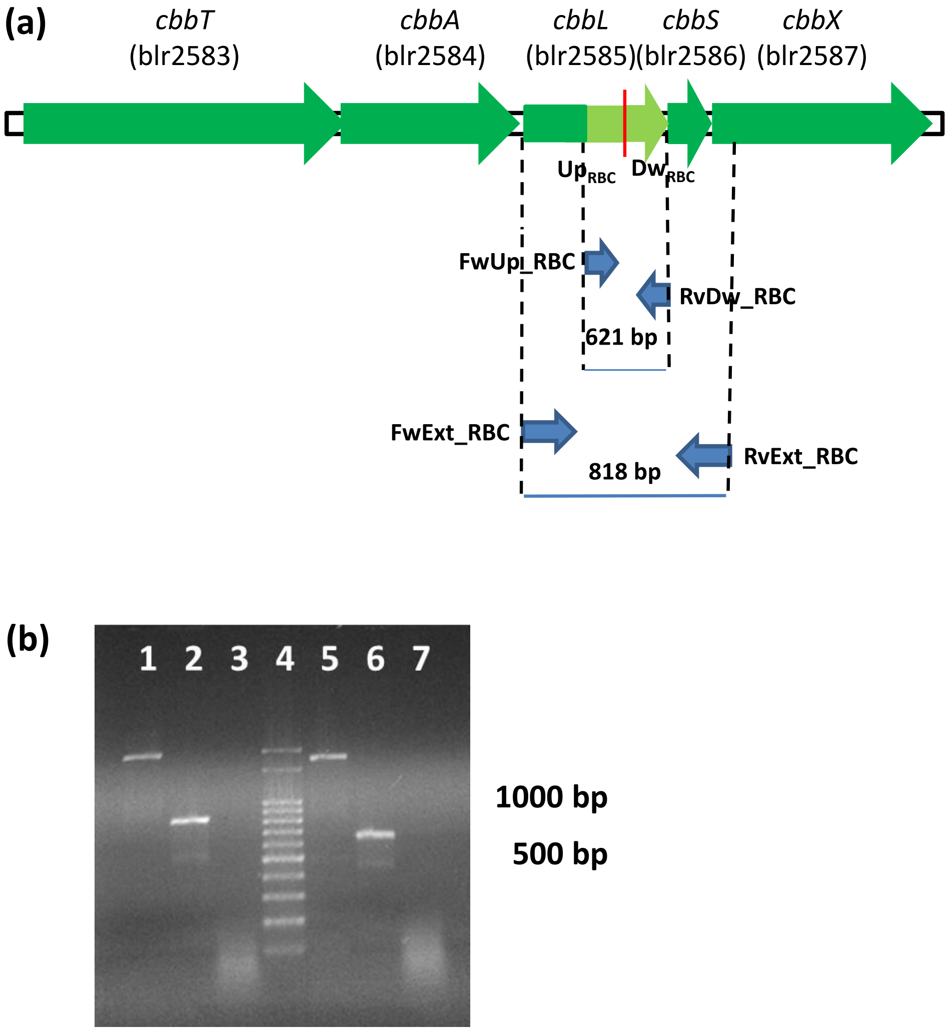

Figure S7 Double crossing-over insertion of the fragment UpDw_RBC into the *B. diazoefficiens* USDA 110 *cbbL* genomic region. (a) Scheme of the insertion, showing the recipient *cbb* gene regions in dark green, the plasmid *cbb* gene regions in light green, the PCR primers in blue, and the *Bam*HI restriction site as a vertical red line. (b) PCR with primers FwExt_RBC and RvExt_RBC (lane 1: USDA 110 wild-type strain; lane 2: Δ*cbbLS* mutant strain; lane 3: negative control), or FwUp_RBC and RvDw_RBC (lane 5: USDA 110 wild-type strain; lane 6: Δ*cbbLS* mutant strain; lane 7: negative control). Lane 4, MW markers, with the positions of the relevant MW marker bands indicated at the right.

(a)

gccgcctagtcattcagagagagaagaacaggagagagtcATGAATGCACATACCGGCACGGTTCGCGGCAAGGAGCGTT
ATCGCTCGGGCGTCATGGAATACAAGCGCATGGGCTATTGGGAGCCCGACTACACGCCAAAGGACACCGACGTCATCGCG
CTGTTCCGCGTCACGCCTCAGGAGGGCGTCGATCCGATCGAGGCGTCCGCTGCGGTTGCCGGCGAGTCCTCGACCGCGAC
CTGGACGGTGGTGTGGACAGATCGCCTGACGGCCGCGGAGAAATATCGCGCGAAATGCTATCGCGTCGATCCGGTGCCGG
GCACGCCGGGCTCGTATTTCGCCTACATCGCCTACGACCTCGACCTGTTCGAGCCGGGCTCGATCGCCAACCTCTCGGCC
TCGATCATCGGCAACGTGTT**TGCCTCGGATCCGTCGACGGC**ATGAAACTGACCCAGGGCTGCTTCTCGTTCCTACCCGAT
CTGACCGACGACCAGATCTATAAGCAGGTGCAGTATTGCCTCGCCAAGGGCTGGGCGGTGAACATCGAGTACACTGACGA
TCCGCATCCCCGCAACACCTATTGGGCGGAGTGCCGCAGGGTGTATGGCGACCGCTACATCCGCATCAGTGGCTTCGATT
CCAGCCAGATGTGGGGCCTGCCGATGTTCGACCTCCAGGACGCCGCCGGCGTGATGATGGAGCTCGCTGGCTGGGAGTCG
GTGCGAATCTCCTTCCTCGTCAACCGGCCGCCGCAGGAAGCCGAATTCGAGCTGGTGCGCCAGGAAGTGGGGGGACGCGC
AATCCGCTACACCACCGT

(b)

atg	aat	gca	cat	acc	ggc	acg	gtt	cgc	ggc	aag	gag	cgt	tat	cgc	tcg	ggc	gtc	atg	gaa
M	N	A	H	T	G	T	V	R	G	K	E	R	Y	R	S	G	V	M	E
tac	aag	cgc	atg	ggc	tat	tgg	gag	ccc	gac	tac	acg	cca	aag	gac	acc	gac	gtc	atc	gcg
Y	K	R	M	G	Y	W	E	P	D	Y	T	P	K	D	T	D	V	I	A
ctg	ttc	cgc	gtc	acg	cct	cag	gag	ggc	gtc	gat	ccg	atc	gag	gcg	tcc	gct	gcg	gtt	gcc
L	F	R	V	T	P	Q	E	G	V	D	P	I	E	A	S	A	A	V	A
ggc	gag	tcc	tcg	acc	gcg	acc	tgg	acg	gtg	gtg	tgg	aca	gat	cgc	ctg	acg	gcc	gcg	gag
G	E	S	S	T	A	T	W	T	V	V	W	T	D	R	L	T	A	A	E
aaa	tat	cgc	gcg	aaa	tgc	tat	cgc	gtc	gat	ccg	gtg	ccg	ggc	acg	ccg	ggc	tcg	tat	ttc
K	Y	R	A	K	C	Y	R	V	D	P	V	P	G	T	P	G	S	Y	F
gcc	tac	atc	gcc	tac	gac	ctc	gac	ctg	ttc	gag	ccg	ggc	tcg	atc	gcc	aac	ctc	tcg	gcc
A	Y	I	A	Y	D	L	D	L	F	E	P	G	S	I	A	N	L	S	A
tcg	atc	atc	ggc	aac	gtg	ttt	gcc	tcg	gat	ccg	tcg	acg	gca	tga	aac	tga	ccc	agg	gct
S	I	I	G	N	V	F	A	S	D	P	S	T	A	-	N	-	P	R	A
gct	tct	cgt	tcc	tac	ccg	atc	tga	ccg	acg	acc	aga	tct	ata	agc	agg	tgc	agt	att	gcc
A	S	R	S	Y	P	I	-	P	T	T	R	S	I	S	R	C	S	I	A
tcg	cca	agg	gct	ggg	cgg	tga	aca	tcg	agt	aca	ctg	acg	atc	cgc	atc	ccc	gca	aca	cct
S	P	R	A	G	R	-	T	S	S	T	L	T	I	R	I	P	A	T	P
att	ggg	cgg	agt	gcc	gca	ggg	tgt	atg	gcg	acc	gct	aca	tcc	gca	tca	gtg	gct	tcg	att
I	G	R	S	A	A	G	C	M	A	T	A	T	S	A	S	V	A	S	I
cca	gcc	aga	tgt	ggg	gcc	tgc	cga	tgt	tcg	acc	tcc	agg	acg	ccg	ccg	gcg	tga	tga	tgg
P	A	R	C	G	A	C	R	C	S	T	S	R	T	P	P	A	-	-	W
agc	tcg	ctg	gct	ggg	agt	cgg	tgc	gaa	tct	cct	tcc	tcg	tca	acc	ggc	cgc	cgc	agg	aag
S	S	L	A	G	S	R	C	E	S	P	S	S	S	T	G	R	R	R	K
ccg	aat	tcg	agc	tgg	tgc	gcc	agg	aag	tgg	ggg	gac	gcg	caa	tcc	gct	aca	cca	ccg	
P	N	S	S	W	C	A	R	K	W	G	D	A	Q	S	A	T	P	P	

Figure S8 DNA and protein sequences of the Δ*cbbLS* mutation. (a) Nucleotide sequence, with the remaining *cbbL* sequence in light blue, the *cbbS* sequence in black, the FwExt_RBC and RvExt_RBC primers shadowed in blue, th FwUp_RBC and RvDw_RBC primers shadowed in black, the complementary region of primers RvUp_RBC and FwDw_RBC in red, the *Bam*HI site generated at this region shadowed in green, and the stop codon introduced at the 5' of *cbbS* shadowed in yellow. The non-coding 5' region is in lowercase. (b) The translated amino acids sequence, with the *Bam*HI site shadowed in green, the stop codon at the N-terminus of CbbS shadowed in yellow, and the additional new stop codons introduced by the frameshift shadowed in light blue. Translation was done with the ExPASy translate tool https://web.expasy.org/translate/.
